# The Prevention of Dental Caries and Oral Sepsis

**Published:** 1912-10-15

**Authors:** 


THE PREVENTION OF DENTAL CARIES AND ORAL SEPSIS. This is the title under which Dr. H. P. Pickerill, who is Professor of Dentistry in the University of Otago, N. Z., treats most scientifically the subject of dental caries in all its important aspects. He gives a concise account of caries and the methods used for its prevention in all times, and presents all that we know of the subject at this time. To this he has added the results of over six years of concentrated investigation of the various phases of the subject. No better or more complete statement of our present knowledge can be found anywhere. The book is charmingly written and any one having an inclination to study this problem will find himself convinced by the excellent work done by the author, who presents his material in a purely scientific manner, citing his authorities when he quotes and giving in sufficient detail his methods for his own findings. The book is a systematic and timely presentation of a subject that is of deep interest just now to all dentists as well as our scientists, and it will be consulted as an authority and have a long life. We have not space to give even a synoptic statement of its contents, or to comment on its material. There are some departures from the accepted theories of the nature of tooth tissues, and the methods by which they suffer pathologically that we would not care to criticise without investigating the authors findings. For instance, he does not find that the bacterial plague is an essential factor in caries, but that the carbohydrate foods are more responsible causes. His ideas as to the place accorded the saliva in causing and restraining caries are not those commonly accepted in this country, and especially his dietetic conclusions with reference to food influences on the salivary functions.

The book brings up for re-discussion many of our accepted theories and we have no doubt it will be the subject of much comment and future investigations, as the author makes it very evident that the last word has not been said by any means as to the cause and prevention of carries. We therefor welcome this contribution as a stimulus to research and further thinking on the most important department of dentistry at the present time. Every thoughtful practitioner should own and study this book carefully. It is printed in London by Bailliere, Lindall and Cox. The S. S. White Dental Manufacturing Co. is the American representative, and we doubt not it may be had through all dental depots.

